# Management strategies for Docking Site refractures: a comparative analysis of 19 patient cases

**DOI:** 10.1186/s13018-024-04938-y

**Published:** 2024-07-25

**Authors:** Yimurang Hamiti, Patiman Abudureyimu, Gang Lyu, Aihemaitijiang Yusufu, Maimaiaili Yushan

**Affiliations:** 1 Department of Microrepair and Reconstructive Surgery, The First Affiliated Hospital of Xinjiang Medical University, Urumqi, Xinjiang P.R. China; 2 Imaging Center of the First Affiliated Hospital of Xinjiang Medical University, Urumqi, Xinjiang P.R. China; 3 Department of Orthopedic Surgery, The Fourth Affiliated Hospital of Xinjiang Medical University, Traditional Chinese Medicine Hospital of Xinjiang Uyghur Autonomous Region, Urumqi, Xinjiang P.R. China

**Keywords:** Docking site, Ilizarov technique, Intramedullary nailing, Percutaneous external plate fixation, Refracture

## Abstract

**Aims:**

This study aimed to compare the clinical effectiveness of intramedullary nailing (IMN), percutaneous external plate fixation (PEPF), and re-applied external fixation (REF) in the treatment of refracture at the consolidated docking site following the removal of external fixation in patients with tibial defects who had previously undergone the Ilizarov bone transport technique.

**Methods:**

A retrospective review was performed on patients who received IMN, PEPF, or REF for refracture at the consolidated docking site subsequent to the removal of external fixation. A collection of data was made regarding the following parameters: age, gender, defect size, treatment methods, external fixation time (EFT), external fixation index (EFI), time of refracture (TOR) subsequent to fixation removal, and docking reunion time (DRT). Bone and functional outcomes were evaluated by the Association for the Study and Application of the Method of Ilizarov (ASAMI) scoring system and the Lower Extremity Functional Scale (LEFS) questionnaire.

**Results:**

The study included 14 males and 5 females with an average age of 38.1 ± 8.9 years (range, 26 to 55 years). Etiologies included post-traumatic osteomyelitis in 11 cases and post-traumatic bone loss in 8 cases. The median bone defect was 5.11 ± 0.87 cm (range, 3.8 to 6.8 cm). Following docking site refracture, 6 cases were treated with IMN, 8 with PEPF, and 5 with REF. All patients achieved both satisfactory bone union and functional outcomes, and there was no significant difference in preoperative baseline data or postoperative outcomes among the three groups.

**Conclusion:**

IMN, PEPF, and REF were all demonstrated favorable postoperative bone and functional outcomes, suggesting their reliability as treatment options for managing docking site refracture following external fixation removal.

## Introduction

Bone transport utilizing the Ilizarov technique has emerged as an effective approach for addressing segmental bone defects, notwithstanding its extended treatment duration and notable complications, such as pin tract infections, docking site complications, shortening, and deformities during the treatment period [1–6]. The docking site represents a pivotal element of the standard bone transport technique, signifying the point at which the transported bone segment reaches its intended location upon completion of the procedure. A critical criterion for the removal of external fixation is the achievement of docking site union and regenerate consolidation, which is defined as the fusion of three out of four cortices observed on both anteroposterior (AP) and lateral radiographs at both the corticotomy (regenerate) and docking sites [2, 7]. Without timely assessment and appropriate management, issues pertaining to the regenerate site or docking union may protract the external fixation duration and ultimately lead to treatment failure.

Docking site refracture post external fixation removal is a significant complication that often necessitates further surgical intervention, thereby extending the treatment duration [3]. Even with thorough radiographic assessment by skilled surgeons, some cases still exhibit refracture at the docking site. Previous systematic reviews and meta-analyses have reported docking site refracture rates of 4% and 5%, respectively [4, 5]. The refracture risk escalates to 3.7 times higher in the reconstruction of tibial defects exceeding 8 cm [5]. Refractures can occur at either the regenerate or docking site, often due to premature fixator removal based on improper union assessment. Treatment approaches include bone grafting at the docking site with internal plate fixation, repeated external fixation, or casting. Regrettably, these aforementioned methods are plagued by issues such as poor bone quality or quantity, excessive periosteal stripping, and prolonged external fixator placement, all of which can adversely impact bone healing and result in significant patient discomfort [1, 3–16].

Given the complexity of managing docking site refractures and the potential drawbacks of current treatment options, it is essential to explore alternative approaches that offer favorable clinical outcomes. In light of this, the objective of this retrospective observational study is to comprehensively evaluate and compare the clinical effectiveness of IMN, PEPF, and REF in addressing docking site refractures following the removal of external fixation. Through a rigorous analysis of patient outcomes, we aim to provide valuable insights into the optimal management strategy for this challenging complication in orthopedic surgery.

## Materials and methods

### Study design and patient selection

This retrospective study was conducted at a university hospital under academic supervision, focusing on patients with tibial defects treated via the Ilizarov bone transport method. Specifically, the study encompassed cases of docking site refractures following external fixation removal, treated either by IMN, PEPF, or REF between January 2008 and June 2020. We excluded patients at the extremes of age, those with severe systemic diseases, or those lacking comprehensive follow-up data. Among 316 cases managed with the Ilizarov technique, 19 met our inclusion criteria, indicating a docking site refracture rate of 6%. Detailed patient data, including demographic information, defect size, treatment approach, EFT, EFI, TOR, and DRT were meticulously documented with demographic data extracted from electronic medical records. Informed consent was obtained from all participants.

### Surgical procedure

All surgical procedures were conducted by a consistent surgical team, adhering to aseptic methods and facilitated by C-arm fluoroscopy. The use of a distractor and intraoperative radiographs was integral for the alignment correction and deformity management during reconstructive surgery. To evaluate the suitability for intramedullary nailing, percutaneous plate placement, or re-applied external fixation, preoperative assessments including physical exams, clinical laboratory tests, routine radiographs, and computed tomography (CT) were conducted. These evaluations aimed to detect any narrowing or obstruction in the medullary canal.

Intramedullary nailing required navigating through both the regenerate area and the docking site, making it a technically demanding procedure. The standard practice involved reamed intramedullary nailing, where the medullary canal was re-established using a long, rigid, pointed guide pin through the regenerative callus. All canals were reamed to a diameter 1 mm greater than that of the nail before inserting the intramedullary locking nail. Subsequent to nail insertion, nail locking and adjuvant bone grafting were performed.

Percutaneous locking plates of different kinds were utilized based on the location of the fracture in patients with a narrowed marrow cavity or possible intramedullary infection. The plate must be long enough to span beyond the distracted region, and previous external fixator screw tracks must be avoided. Insertion through or next to such a position is associated with the risk of fracture. After the plate was placed, the proximal and distal ends were secured with 3 or 4 screws. Additional bone grafting was performed around the docking site in all patients to facilitate docking union. The wounds were closed once the fracture fixation was completed and the final radiological examination of all fixation components was conducted.

In cases where intramedullary nailing or plate placement was unsuitable, particularly due to medullary canal conditions, poor soft tissue coverage, or the need for dynamic adjustments, REF was employed. For the re-applied external fixation, the procedure entailed the reapplication of an external fixator following the same principles as the initial application. The process required meticulous positioning of pins and wires to guarantee the stability and proper alignment of the bone fragments, thereby ensuring the stability of the treated region and the preservation of the surrounding tissues.

### Postoperative management

Commencing from the first postoperative day, patients were encouraged to engage in active knee, ankle, and foot exercises to promote functional recovery. Clinical and radiological evaluations were conducted on a monthly basis to monitor progress. The initiation of partial weight-bearing was recommended immediately post-surgery, with gradual increases tailored based on radiological evidence of healing. Rigorous follow-up was maintained for all patients, ensuring comprehensive data collection for final analysis during the last clinical visit. The criteria for removal of plate or external fixation were stringently based on the assessment of both bone and functional outcomes. Bone union was specifically defined by the presence of a visible callus bridging at least three out of four cortices at the fracture site, as confirmed by radiological examination. Upon successful confirmation of bone union and fixation removal, each patient was provided with long leg casting for a minimum duration of 4–6 weeks to ensure adequate support and stabilization during the critical early phase of recovery post-fixation removal.

### Outcome evaluation

Digital medical records were used for the evaluation of the following outcome measures, and hospital picture archiving and communication system images were used for the visualization of the results: DRT, postoperative LEFS, and ASAMI classification. Participants’ bone and functional outcomes were graded according to the ASAMI score [6]. Additionally, patients completed the LEFS questionnaire, comprising 20 items related to daily activities like walking and squatting, to gauge functional status. The LEFS, with a maximum score of 80 (indicating superior functional ability), is recognized for its robustness and reliability in evaluating lower extremity function, as evidenced by its application in multiple research studies [17].

### Statistical analysis

Statistical analysis was performed using SPSS 25.0 software (SPSS software, Chicago, IL, USA). The obtained data were first tested for normal distribution. Continuous variables are presented as the mean ± standard deviation while proportions are presented for categorical variables. Kruskal–Wallis tests were performed to compare the differences between the groups as appropriate. Categorical data were evaluated with Fisher’s exact test. A *P*-value of < 0.05 indicated a statistically significant difference.

## Results

This study encompassed 19 patients, comprising 14 males and 5 females, with an average age of 38.1 ± 8.9 years (range, 26 to 55 years). The underlying etiologies were post-traumatic osteomyelitis (11 cases) and post-traumatic bone loss (8 cases). Bone defect lengths at the initial surgery ranged from 3.8 to 6.8 cm, with a median of 5.11 ± 0.87 cm. Bifocal bone transport was employed in 15 cases, and trifocal transport in 4 cases. The average EFT and EFI were 7.25 ± 1.37 months (range, 4.2–9.2 months) and 1.48 ± 0.31 months/cm (range, 0.7–1.8 months/cm), respectively. The TOR ranged from 3.4 to 8.6 months, with an average of 5.65 ± 1.25 months. Treatment modalities included IMN in 6 cases, PEPF in 8 cases, and REF in 5 cases. A comparative analysis of demographic and preoperative baseline data is detailed in Table 1, revealing no significant differences between the groups (*P* > 0.05). Clinical and radiographic union was achieved in all patients, with an average DRT was 6.27 ± 0.32 months (range, 5.8–6.9 months). The mean LEFS score was 73.9 ± 3.6 points (range, 68–78 points). The above results were summarized in Table 2. Statistical analysis showed no significant differences between the observed outcomes.

**Table 1:** Comparison of the demographic and preoperative baseline data

Parameter	IMN	PEPF	REF	*P*-value
Mean age (years)	35.3 ± 7.0	38.3 ± 8.7	41.2 ± 12.0	0.752
Mean defect size (cm)	4.78 ± 0.70	5.55 ± 1.00	4.82 ± 0.67	0.177
Mean EFT (months)	7.10 ± 1.16	7.71 ± 1.60	6.70 ± 1.22	0.200
Mean EFI (months/cm)	1.55 ± 0.38	1.43 ± 0.33	1.50 ± 0.24	0.379
Mean TOR (months)	5.58 ± 2.08	5.73 ± 0.88	5.60 ± 0.50	0.756

**IMN** intramedullary nailing, **PEPF** percutaneous external plate fixation, **REF** re-applied external fixation, **EFT** external fixation time, **EFI** external fixation index, **TOR** time of refracture

**Table 2:** Comparison of the postoperative outcomes

Parameter	IMN	PEPF	REF	*P*-value
Mean DRT (months)	6.28 ± 0.29	6.39 ± 0.37	6.08 ± 0.23	0.216
LEFS	74.5 ± 3.5	74.3 ± 3.6	72.8 ± 4.1	0.750

**IMN** intramedullary nailing, **PEPF** percutaneous external plate fixation, **REF** re-applied external fixation, **DRT** docking reunion time, **LEFS** Lower Extremity Functional Scale questionnaire

**Fig. 1:**
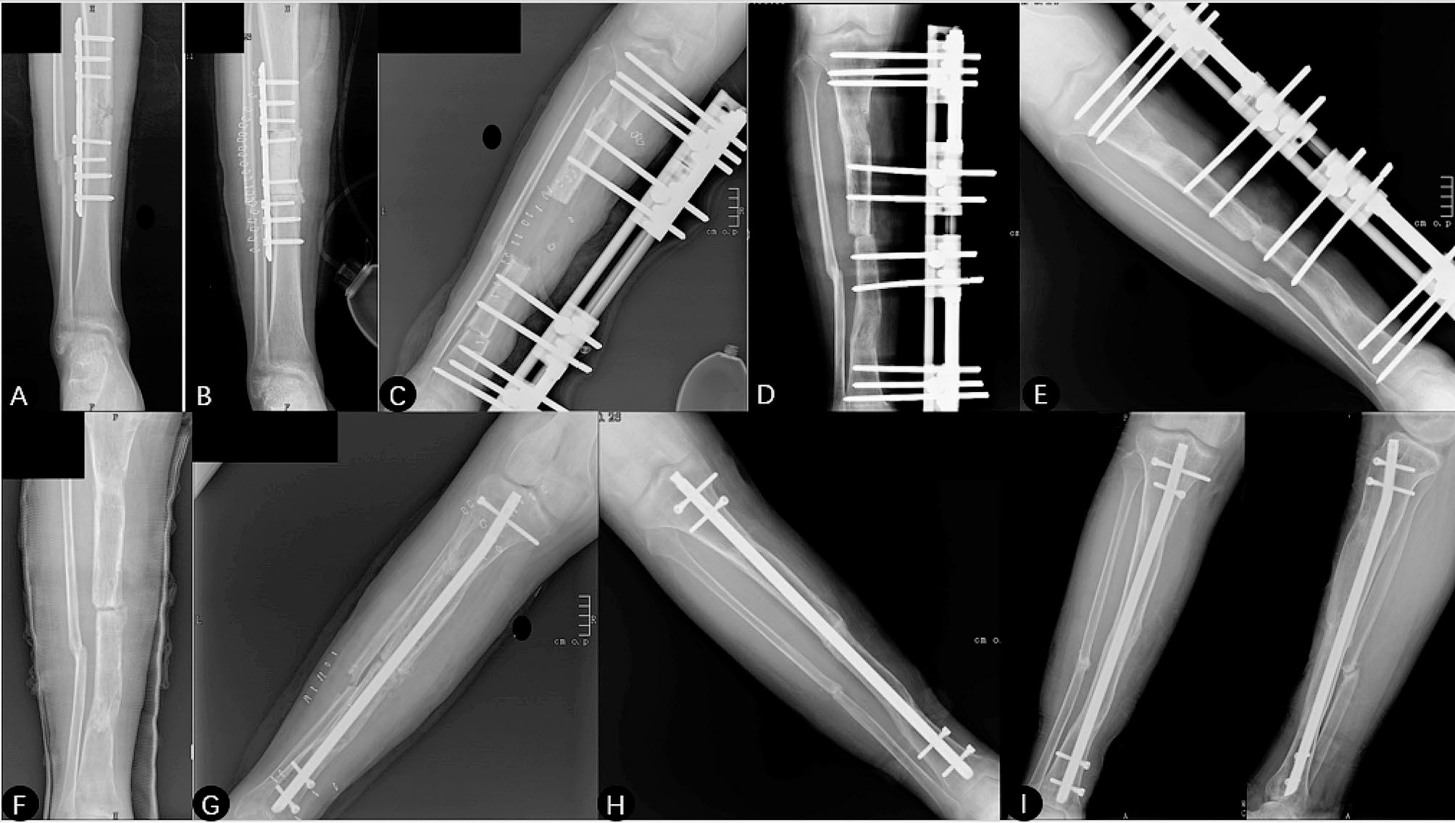
A 32-year-old male presented with fracture-related infection of the right tibia, which developed into chronic osteomyelitis, and underwent Trifocal bone transport using the Ilizarov technique. Docking site refracture was encountered 5.6 months after the removal of the external fixator, which was managed by Intramedullary nailing along with autologous bone grafting. **(A)** Anteroposterior radiograph showed nonunion and fracture-related infection after open reduction and internal fixation. **(B)** Anteroposterior radiograph showed chronic osteomyelitis after nonunion was managed by autologous bone grafting. **(C)** Anteroposterior radiograph on the first postoperative day after application of trifocal bone transport using the Ilizarov technique. **(D)** Anteroposterior radiograph during bone transport. **(E)** Anteroposterior radiograph before removal of external fixation after consolidated docking union. **(F)** The docking site refracture was encountered 6.6 months after the removal of the fixator and was managed temporarily by casting. **(G)** Anteroposterior radiograph demonstrated docking site refracture managed by intramedullary nailing. **(H)** Anteroposterior radiographs at 3-month follow-up. **(I)** Anteroposterior radiograph at 6.6 month follow-up time showed union at the docking site

**Fig. 2:**
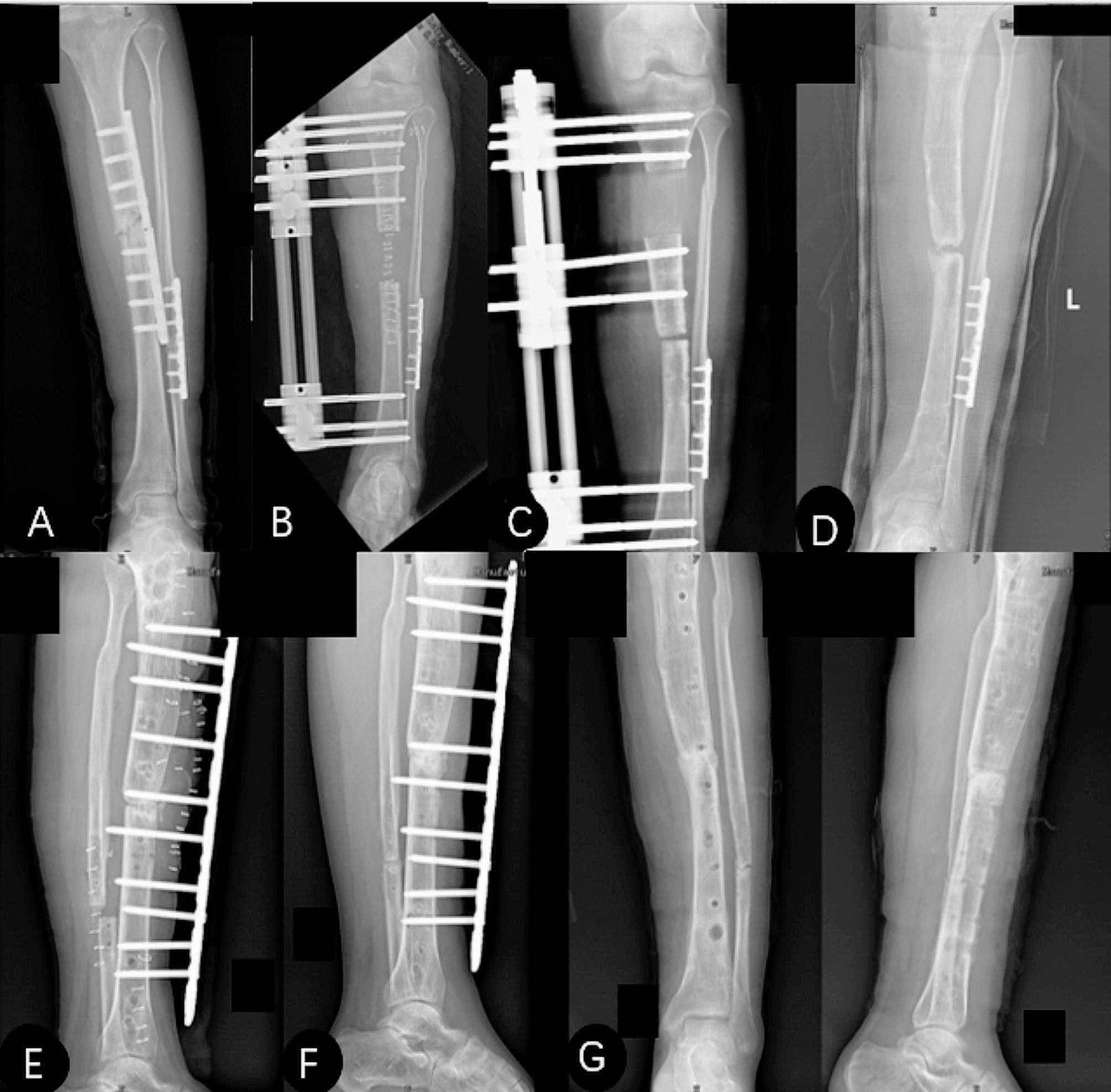
A 47-year-old female present with post-traumatic osteomyelitis of the left tibia underwent Bifocal bone transport using the Ilizarov technique. Docking site refracture was encountered 4.2 months after the removal of the external fixator, which was managed by percutaneous plate external fixation along with autologous bone grafting. **(A)** Anteroposterior radiograph showed plate refracture and fracture-related infection after open reduction and internal fixation. **(B)** Anteroposterior radiograph on the first postoperative day after application of bifocal bone transport using the Ilizarov technique. **(C)** Anteroposterior radiograph during bone transport. **(D)** The docking site refracture was encountered 4.2 months after the removal of the fixator and was managed temporarily by casting. **(E)** Anteroposterior radiograph demonstrated docking site refracture managed by percutaneous plate external fixation along with autologous bone grafting. **(F)** Anteroposterior radiographs at 6.8 months follow-up showed union at the docking site. **(G)** Anteroposterior radiograph at last clinical visit after removal of percutaneous plate external fixation

Bone and functional outcomes, evaluated using the ASAMI criteria, are presented in Table 3. In the IMN group, bone outcomes were rated as excellent (66.7%), good (16.7%), and fair (16.7%), with functional outcomes classified as excellent (83.3%) and good (16.7%). In the PEPF group, bone outcomes were excellent (62.5%), good (25.0%), and fair (12.5%), and functional outcomes were excellent (75.0%) and good (25.0%). In the REF group, bone outcomes were excellent (80.0%) and good (20.0%), with functional outcomes similarly rated as excellent (80.0%) and good (20.0%). All three treatment groups demonstrated satisfactory bone and functional outcomes with no significant disparities. No limb shortening or development of persistent deformity was associated with any of the refractures at the docking site. No instances of limb shortening or persistent deformity were associated with any refractures at the docking site. At the final follow-up, significant complications such as malunion or nonunion at the docking site, internal fixation failure, iatrogenic neurological paralysis, or voluntary amputations were not observed. Further patient details are illustrated in Figs. 1 and 2.

**Table 3:** Comparison of the bone and functional results according ASAMI classification

Outcomes	Treament	Numbers/Percentage			*P*-value
		Excellent	Good	Fair	
Bone results	IMN	4 (66.7%)	1 (16.7%)	1 (16.7%)	0.760
	PEPF	5 (62.5%)	2 (25.0%)	1 (12.5%)	
	REF	4 (80.0%)	1 (20.0%)	0	
Functional results	IMN	5 (83.3%)	1 (16.7%)	0	0.932
	PEPF	6 (75.0%)	2 (25.0%)	0	
	REF	4 (80.0%)	1 (20.0%)	0	

**IMN** intramedullary nailing, **PEPF** percutaneous external plate fixation, **REF** re-applied external fixation

## Discussion

This retrospective comparative study evaluated the clinical efficacy of IMN, PEPF, and REF in managing consolidated docking site refractures post external fixation removal. A key finding is that all treatment modalities resulted in satisfactory outcomes, both subjectively and objectively, suggesting these approaches as viable options in appropriate cases. To the best of our knowledge, this is the first paper that investigates the comparative effectiveness of REF, IMN, and PEPF in this context.

As extensively documented in the literature, managing segmental bone defects poses a considerable challenge for orthopedic surgeons, primarily due to the profound impact on patients’ quality of life and the lack of a standardized reconstruction treatment protocol. These factors collectively contribute to various clinical obstacles, potentially affecting outcomes adversely [1–6, 8–16]. The Ilizarov bone transport technique, rooted in distraction osteogenesis, has emerged as a widely accepted and time-tested method for treating bone defects [1, 3, 6–12]. Notably, patients with segmental tibial defects who undergo Ilizarov bone transport often express a keen desire to have the external frame removed. However, premature or improper removal of the fixator can lead to several unforeseen complications. These include deformity, limb shortening, nonunion at the docking site, and refractures either at the regenerate site or the docking site itself [1, 4, 5].

The issue of problematic docking sites has been well-documented in several studies. Cierny et al. [8] analyzed 21 patients with tibial defects treated using the bone transport technique, identifying two instances of pseudoarthrosis at the docking site. Similarly, Paley et al. [9] reported on 19 patients with tibial defects, of which seven required debridement and bone grafting at the docking site for consolidation. Additionally, three of these patients underwent debridement at the docking site exclusively to eliminate fibrocartilage at the bone ends. In a study employing double-level bone transport, Zhang et al. [10] treated 16 patients with extensive post-traumatic tibial bone defects. Of these, two patients presented with nonunion at the docking site, which was addressed through the excision of invaginated soft tissue, debridement, and the application of autogenous bone grafts. These reports underscore the complexity and the need for individualized management strategies at the docking site to ensure successful bone defect reconstruction. Notably, bone transport with hybrid techniques, combines the advantages of external fixators with internal implants, allowing for the early removal of the external fixator after the transport phase is over, which can significantly shorten the EFT. The internal implants maintain bony alignment and stability, protecting the distraction callus and docking site from fracture and deformity. Various hybrid techniques differ mainly in the timing of internal implant insertion, such as sequential internal fixation after bone transport or simultaneous internal fixation with bone transport. One such hybrid technique is nailing after bone transport [18], which transitions from the external fixator to internal nailing immediately after the transport phase, avoiding a pin holiday. Additional fixation of the transported segment is achieved through a predrilled hole in the nail to stabilize the docking site. This method requires careful planning and technical precision to ensure a seamless transition from external to internal fixation, minimizing risks such as contamination or deep infection. By maintaining stability and protecting the docking site from fractures and deformities, these hybrid techniques can significantly mitigate the risk of docking site consolidation failure. While these methods can improve outcomes, their routine use should be contingent upon the availability of expertise and resources, as well as the specific clinical circumstances of each patient. When executed correctly, hybrid techniques offer a robust solution for the challenges associated with docking site consolidation.

The incidence of refractures at the docking site represents a significant concern in orthopedic surgery. Systematic reviews and meta-analyses have estimated the refracture rates at docking sites to be approximately 4% and 5%, respectively [4, 5]. Highlighting this issue, Paley et al. [9] observed a case among 19 patients where docking site refracture necessitated surgical intervention, specifically the reapplication of the Ilizarov apparatus. In a larger cohort, McNally et al. [11] treated 79 patients using the Ilizarov bone transport technique, with 8 patients (10.1%) experiencing refractures approximately 10.1 months post-frame removal. These cases were predominantly managed through revision debridement and repeated frame fixation (three cases), cast bracing (two cases), intramedullary nailing (one case), and plate fixation (one case). Additionally, Xu et al. [12] conducted a study on 31 patients with extensive tibial bone and soft tissue defects, treated via the trifocal bone transport technique. In their series, one patient encountered a refracture at the docking site post-frame removal, which was addressed using internal plate fixation and autologous bone grafting. These findings underscore the need for careful management and follow-up post-frame removal to mitigate the risk of refractures at the docking site.

In scenarios where standardized treatment protocols are lacking, orthopedic surgeons often rely on their clinical judgment to make decisions. This approach, however, is associated with various risks, particularly when dealing with poor bone quality or quantity, excessive periosteal stripping, intraoperative soft tissue damage, and the prolonged use of external fixators [1, 3–16]. To mitigate the risks associated with these current methods, a combination of bone grafting with intramedullary nailing or percutaneous locking plate fixation could be considered. These approaches may potentially reduce the occurrence of complications related to the methods currently in use.

Intramedullary nailing (IMN) has been established as the standard treatment for diaphyseal tibia fractures, contrasting with other operational treatments like internal plate fixation. IMN often allows for reaming with minimal soft tissue dissection, dependent on fracture location and deformity. Additionally, combining IMN with bone grafting can address issues of delayed union or nonunion, enhance patient comfort, enable earlier weight-bearing, and reduce the frequency of complications [13, 14, 19–22]. However, the technical feasibility of IMN in managing docking site refractures is limited, especially in cases with a narrow marrow cavity. In scenarios involving infection, the application of IMN and bone grafting requires cautious execution due to the heightened risk of reinfection. Studies have shown a higher recurrence of infection in patients undergoing internal fixation for infected long-bone nonunions as opposed to external fixation [23]. The risk of exacerbating intramedullary infections is particularly pertinent with IMN, potentially leading to advanced osteomyelitis (Mader–Cierny type IV) [15, 24, 25]. Previous research has shown that the regenerated segment of the tibia does not have a medullary cavity until at least two years after the frame is removed. After the frame is taken off, the medullary cavity remains blocked at the docking site for at least 43 months [26]. Thus, the cross-sectional images using computed tomography is recommended to evaluate the actual condition at the regenerated area before application of IM in the management of docking site refracture.

In such instances, PEPF presents itself as a viable alternative. PEPF offers a simpler assembly and disassembly process compared to IMN and is associated with a lower incidence of deep infection [15]. Additionally, PEPF is advantageous in terms of patient compliance, as it is generally less cumbersome than other fixation methods. This is particularly relevant considering the psychological impact and other complications associated with prolonged use of the bone transport technique and external fixators [27, 28]. Thus, PEPF should be considered a significant alternative in cases where IMN is unsuitable, especially in the context of narrow marrow cavities or existing infections.

Despite the effectiveness of IMN and PEPF, REF presents itself as a compelling alternative in scenarios, particularly in situations where IMN or PEPF may not be suitable due to various patient-specific factors like bone quality, soft tissue condition, or the complexity of the fracture. REF offers the advantage of being less invasive and more adaptable to individual patient anatomy and healing progress. Its application is especially pertinent in cases where there’s a need for dynamic adjustment, or when other methods pose a higher risk due to the patient’s overall health status or the specific nature of the bone defect.

The utility of routine bone grafting at the docking site in orthopedic surgery remains a subject of ongoing debate. Giotakis et al. [2] posited that bone grafting might not be imperative if the clearance and preparation of the docking site result in two coapted surfaces with substantial contact area. Paley et al. [29] reported that all 25 patients with tibial nonunion and bone defects achieved bone union at the docking sites with only compression and no bone grafts. Xu et al. [12] concluded an increased surface area at the bone ends could bolster docking site stability by diminishing shear forces, thus facilitating union without the need for grafting. Contrastingly, in our current series, all patients received additional bone grafting at the docking site to promote docking union. This approach underscores the notion that treatment duration could potentially be extended if adequate focus is not placed on optimally managing the docking site. The divergence in practices highlights the need for a more nuanced understanding of the conditions under which bone grafting is beneficial, underscoring the importance of individualized patient assessment in determining the most effective treatment strategy.

There are limitations to our study that must be acknowledged. This is a retrospective study with inherent limitations, including selection and indication biases. The primary limitation of our study is the relatively small sample size. While our findings provide valuable insights into the treatment of refractures at the docking site using IMN, PEPF, and REF, the limited number of cases may affect the generalizability of the results. Future studies with larger sample sizes are necessary to validate these findings and to provide a more comprehensive understanding of the efficacy and safety of these treatment modalities. Additionally, a larger cohort would allow for more robust statistical analyses and the identification of potential subgroups that may benefit more from specific treatments.

## Conclusion

In conclusion, our findings indicate that refractures at the docking site can be successfully treated with IMN, PEPF, and REF in centers with specialized orthopedic expertise. IMN is generally preferred for patients without a history of septic medullary implants, PEPF serves as a viable option for patients with mature regenerated consolidation and offers a reduced risk of reinfection. REF emerges as a alternative in cases where IMN or PEPF may be less suitable due to patient-specific factors such as bone quality, soft tissue condition, or the complexity of the fracture. Nonetheless, the small sample size of our study limits the robustness of our conclusions. To validate and expand upon our findings, larger-scale studies are required. Future research should aim to include a more diverse patient population and provide more detailed data on long-term outcomes. Despite this limitation, our study highlights the importance of individualized treatment plans and comprehensive clinical assessments to optimize the management of docking site refractures.

## Data Availability

No datasets were generated or analysed during the current study.

## References

[1] Borzunov DY, Kolchin SN, Malkova TA. Role of the Ilizarov non-free bone plasty in the management of long bone defects and nonunion: problems solved and unsolved. World J Orthop. 2020;11:304–18. 32572367 10.5312/wjo.v11.i6.304 PMC7298454

[2] Giotakis N, Narayan B, Nayagam S. Distraction osteogenesis and nonunion of the docking site: is there an ideal treatment option? Injury. 2007;38(Suppl 1):S100–107. 17383479 10.1016/j.injury.2007.02.015

[3] Paley D. Problems, obstacles, and complications of limb lengthening by the Ilizarov technique. Clin Orthop Relat Res. 1990;81–104. 2403498

[4] Yin P, Ji Q, Li T, Li J, Li Z, Liu J, et al. A systematic review and Meta-analysis of Ilizarov methods in the treatment of infected Nonunion of Tibia and Femur. PLoS ONE. 2015;10:e0141973. 26529606 10.1371/journal.pone.0141973 PMC4631548

[5] Papakostidis C, Bhandari M, Giannoudis PV. Distraction osteogenesis in the treatment of long bone defects of the lower limbs: effectiveness, complications and clinical results; a systematic review and meta-analysis. Bone Joint J. 2013;95–B:1673–80. 24293599 10.1302/0301-620X.95B12.32385

[6] Borzunov DY. Long bone reconstruction using multilevel lengthening of bone defect fragments. Int Orthop. 2012;36:1695–700. 22581353 10.1007/s00264-012-1562-1 PMC3535043

[7] Fischgrund J, Paley D, Suter C. Variables affecting time to bone healing during limb lengthening. Clin Orthop Relat Res. 1994;31–7. 8156692

[8] Cierny G, Zorn KE. Segmental tibial defects. Comparing conventional and Ilizarov methodologies. Clin Orthop Relat Res. 1994;118–23. 8156662

[9] Paley D, Maar DC. Ilizarov bone transport treatment for tibial defects. J Orthop Trauma. 2000;14:76–85. 10716377 10.1097/00005131-200002000-00002

[10] Zhang Y, Wang Y, Di J, Peng A. Double-level bone transport for large post-traumatic tibial bone defects: a single centre experience of sixteen cases. Int Orthop. 2018;42:1157–64. 29129017 10.1007/s00264-017-3684-y

[11] McNally M, Ferguson J, Kugan R, Stubbs D. Ilizarov Treatment protocols in the management of infected Nonunion of the Tibia. J Orthop Trauma. 2017;31(Suppl 5):S47–54. 28938393 10.1097/BOT.0000000000000987

[12] Xu Y-Q, Fan X-Y, He X-Q, Wen H-J. Reconstruction of massive tibial bone and soft tissue defects by trifocal bone transport combined with soft tissue distraction: experience from 31 cases. BMC Musculoskelet Disord. 2021;22:34. 33413256 10.1186/s12891-020-03894-y PMC7788851

[13] Richmond J, Colleran K, Borens O, Kloen P, Helfet DL. Nonunions of the distal tibia treated by reamed intramedullary nailing. J Orthop Trauma. 2004;18:603–10. 15448449 10.1097/00005131-200410000-00005

[14] Xu Y, Ma T, Ren C, Li M, Lu Y, Sun L et al. Treatment of tibial large bone defects: A comparative study of bone transport over an intramedullary nail in combination with antibiotic-impregnated calcium sulphate versus bone transport alone with antibiotic-impregnated calcium sulphate. Injury. 2022;S0020-1383(22)00707-0. 10.1016/j.injury.2022.09.042 36192202

[15] Lu Y, Ma T, Ren C, Li Z, Sun L, Xue H, et al. Treatment of segmental tibial defects by bone transport with circular external fixation and a locking plate. J Int Med Res. 2020;48:300060520920407. 32351151 10.1177/0300060520920407 PMC7218946

[16] Kocaoglu M, Eralp L, Rashid HU, Sen C, Bilsel K. Reconstruction of segmental bone defects due to chronic osteomyelitis with use of an external fixator and an intramedullary nail. J Bone Joint Surg Am. 2006;88:2137–45. 17015589 10.2106/JBJS.E.01152

[17] Binkley JM, Stratford PW, Lott SA, Riddle DL. The Lower Extremity Functional Scale (LEFS): scale development, measurement properties, and clinical application. North American Orthopaedic Rehabilitation Research Network. Phys Ther. 1999;79:371–83. 10201543

[18] Quinnan SM, Lawrie C. Optimizing bone defect Reconstruction-Balanced Cable Transport with Circular External fixation. J Orthop Trauma. 2017;31(10):e347–55. 28938286 10.1097/BOT.0000000000000994

[19] Petrisor B, Anderson S, Court-Brown CM. Infection after reamed intramedullary nailing of the tibia: a case series review. J Orthop Trauma. 2005;19:437–41. 16056073 10.1097/01.bot.0000161542.93624.8d

[20] Bong MR, Kummer FJ, Koval KJ, Egol KA. Intramedullary nailing of the lower extremity: biomechanics and biology. J Am Acad Orthop Surg. 2007;15:97–106. 17277256 10.5435/00124635-200702000-00004

[21] Court-Brown CM, McQueen MM, Quaba AA, Christie J. Locked intramedullary nailing of open tibial fractures. J Bone Joint Surg Br. 1991;73:959–64. 1955445 10.1302/0301-620X.73B6.1955445

[22] Högel F, Gerber C, Bühren V, Augat P. Reamed intramedullary nailing of diaphyseal tibial fractures: comparison of compression and non-compression nailing. Eur J Trauma Emerg Surg. 2013;39:73–7. 26814925 10.1007/s00068-012-0237-3

[23] Bose D, Kugan R, Stubbs D, McNally M. Management of infected nonunion of the long bones by a multidisciplinary team. Bone Joint J. 2015;97–B:814–7. 26033062 10.1302/0301-620X.97B6.33276

[24] Gupta S, Malhotra A, Mittal N, Garg SK, Jindal R, Kansay R. The management of infected nonunion of tibia with a segmental defect using simultaneous fixation with a monorail fixator and a locked plate. Bone Joint J. 2018;100–B:1094–9. 30062945 10.1302/0301-620X.100B8.BJJ-2017-1442.R1

[25] Megas P, Saridis A, Kouzelis A, Kallivokas A, Mylonas S, Tyllianakis M. The treatment of infected nonunion of the tibia following intramedullary nailing by the Ilizarov method. Injury. 2010;41:294–9. 20176169 10.1016/j.injury.2009.09.013

[26] Giannikas KA, Bayam L, Naraen A, Buckley J, Maganaris C, Wilkes RA, et al. Cross-sectional anatomy in postdistraction osteogenesis tibia. J Orthop Sci. 2007;12:430–6. 17909927 10.1007/s00776-007-1153-y

[27] Yushan M, Abulaiti A, Maimaiti X, Hamiti Y, Yusufu A. Tetrafocal (three osteotomies) and pentafocal (four osteotomies) bone transport using Ilizarov technique in the treatment of distal tibial defect-preliminary outcomes of 12 cases and a description of the surgical technique. Injury. 2022;53:2880–7. 35691766 10.1016/j.injury.2022.06.006

[28] Hamiti Y, Yushan M, Lu C, Yusufu A. Reconstruction of massive tibial defect caused by osteomyelitis using induced membrane followed by trifocal bone transport technique: a retrospective study and our experience. BMC Surg. 2021;21:419. 34911504 10.1186/s12893-021-01421-x PMC8672610

[29] Paley D, Catagni MA, Argnani F, Villa A, Benedetti GB, Cattaneo R. Ilizarov treatment of tibial nonunions with bone loss. Clin Orthop Relat Res. 1989;146–65. 2924458

